# Chitinase-3-Like Protein 1 (YKL-40) Is a New Biomarker of Inflammation in Psoriasis

**DOI:** 10.1155/2017/9538451

**Published:** 2017-08-28

**Authors:** Joanna Salomon, Łukasz Matusiak, Danuta Nowicka-Suszko, Jacek C. Szepietowski

**Affiliations:** Department of Dermatology, Venereology and Allergology, Wrocław Medical University, Wrocław, Poland

## Abstract

**Purpose:**

The evaluation of new inflammatory biomarkers in psoriasis could determine therapeutic decisions. Chitinase-3-like protein 1 (YKL-40) plays a role in inflammation. The study was undertaken to check whether YKL-40 is a reliable biomarker of inflammation in psoriasis.

**Materials and Methods:**

55 psoriatic patients were enrolled, including 21 men and 34 women, aged from 18 to 88 years. The PASI and body surface area were calculated for all patients. Blood samples were taken to evaluate serum concentration of YKL-40, as well as other inflammatory parameters, including CRP, ESR, white blood cell count, and neutrophil count. The measurements of YKL-40 level were performed by enzyme-linked immunosorbent assay (ELISA).

**Results:**

YKL-40 serum concentration was significantly higher in psoriatic patients than in the control group. No correlations were found between YKL-40 levels and other clinical or laboratory parameters. Serum YKL-40 level was elevated in 81.8% patients, whereas CRP and WBC in 20% and 7.3% of patients, respectively.

**Conclusions:**

YKL-40 could be considered as a useful biomarker of inflammation in psoriasis and is more sensitive than CRP or WBC. Increased YKL-40 may indicate psoriatic patients with a higher level of systemic inflammation, which may determine disease management.

## 1. Introduction

Psoriasis is a common inflammatory skin disorder of complex pathomechanisms, which is genetically conditioned and influenced by environmental factors. The typical cutaneous symptoms are papules and plaques covered with silvery scales. However, the course, severity of symptoms, and progress of the disease are unpredictable and vary in each patient. Recently, psoriasis is no longer considered to be exclusively a skin disorder, since there is considerable evidence supporting the concept of psoriasis as a systemic inflammatory disease [1–3]. Thus, patients with cutaneous psoriasis may suffer from many systemic comorbidities, which are characterized by chronic inflammation of various grades [3]. One of the most frequent complications of psoriasis is arthritis that may affect up to 40% of psoriatic patients [4]. However, it has also been shown that patients with psoriasis are at higher risk of developing cardiovascular disorders, diabetes, metabolic syndrome, inflammatory bowel disease, and many others [3, 5–7]. The severity of psoriasis and the presence of comorbidities influence patients' life expectancy [6]. That is why it is necessary to search for new sensitive biomarkers which could reflect both the activity of psoriasis and the level of systemic inflammation—a parameter difficult to assess objectively. Evaluation of psoriatic patients' inflammatory biomarkers could determine the therapeutic approach.

Chitinase-3-like protein 1 (YKL-40) is one of the 18 glycosyl hydrolases, the conservative family of mammalian chitinases [8]. However, this protein has no enzymatic activity and its biological role still remains unclear. It is known that YKL-40 plays an important role in inflammation, proliferation, and angiogenesis [9, 10]. This protein has already been evaluated in many inflammatory diseases, including inflammatory bowel diseases, rheumatoid arthritis, inflammatory lung diseases, osteoarthritis, viral hepatitis, cardiovascular disease, and many malignant processes [9, 10]. Recently, there have been some reports evaluating the role of YKL-40 in cutaneous psoriasis; however, the results obtained so far are conflicting [11–16]. That is why we decided to explore this matter and check whether YKL-40 may play an important role in pathomechanisms of psoriasis.

## 2. Materials and Methods

The study was performed on a group of 55 patients suffering from psoriasis. The group consisted of 21 women and 34 men, aged from 18 to 88 years (mean 48.56 ± 13.2 years). The disease duration ranged from 0.1 to 60 years (mean 15.56 ± 15.4 years). The assessment of skin lesions was carried out using the Psoriasis Area and Severity Index (PASI). Moreover, the body surface area (BSA) was calculated. The severity of skin lesions measured by the PASI ranged from 3.8 to 32.1 points (mean 10.8 ± 6.8). BSA varied between 2.7 and 80% (mean 19.13 ± 17%). The complete description of psoriatic patients is presented in Table 1. All patients had a negative history of malignancies, neither had symptoms of any infection. However, some of them suffered from comorbidities, which were hypertension (19 patients, 34.5%), diabetes (5 patients, 9%), hyperlipidaemia (5 patients, 9%), and single cases of obstructive pulmonary disease, epilepsy, recurrent gastritis, hypothyreosis, nephrolithiasis, and depression. The control group was made of 37 healthy, nonpsoriatic individuals, not suffering from any significant chronic disorders, specially selected mainly from the blood donors. The control group consisted of 14 men and 23 women, aged from 28 to 71 years (mean 45.5 ± 12.6 years). The psoriatic patients and the control group matched for age (*p* = 0.38), which is particularly important, because the serum level of YKL-40 may depend on age.

Blood samples were taken from all the patients to evaluate serum concentration of YKL-40. Furthermore, measurements of additional inflammatory parameters, including CRP (C-reactive protein), ESR (erythrocyte sedimentation rate), white blood cell count (WBC), and neutrophil count, were performed. For the assessment of serum YKL-40, samples of venous blood were collected, then the serum was separated and kept frozen at the temperature of −70°C until it was analysed. The measurements were performed using enzyme-linked immunosorbent assay (ELISA) by R&D systems, Minneapolis, USA (catalogue number DC3L10), according to the manufacturer's instruction.

The Kolmogrov-Smirnov test was used to evaluate the data distribution. The quantitative variables were presented in the form of medians and ranges. The comparisons between the examined groups were performed by Mann-Whitney *U* test. Correlations between the variables were calculated using Spearman's rank correlation. *p* value less than 0.05 was considered to be statistically significant.

The study was conducted in compliance with ethic regulations and follows the principles of the Declaration of Helsinki. The study has been approved by the Bioethics Committee of the Wrocław Medical University (opinion number 153/2017). The written informed consent was obtained from all participants.

## 3. Results

**Figure 1:**
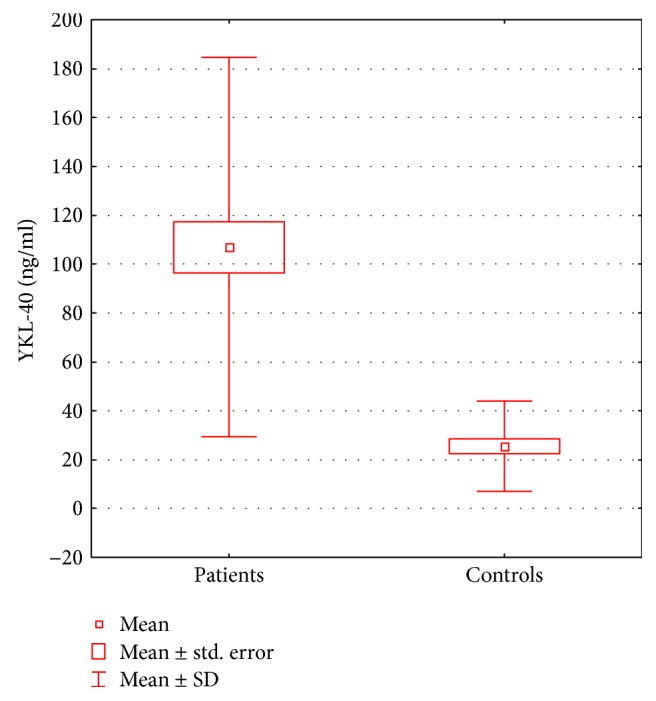
Comparison of serum YKL-40 concentration in patients with psoriasis and the control group.

**Figure 2:**
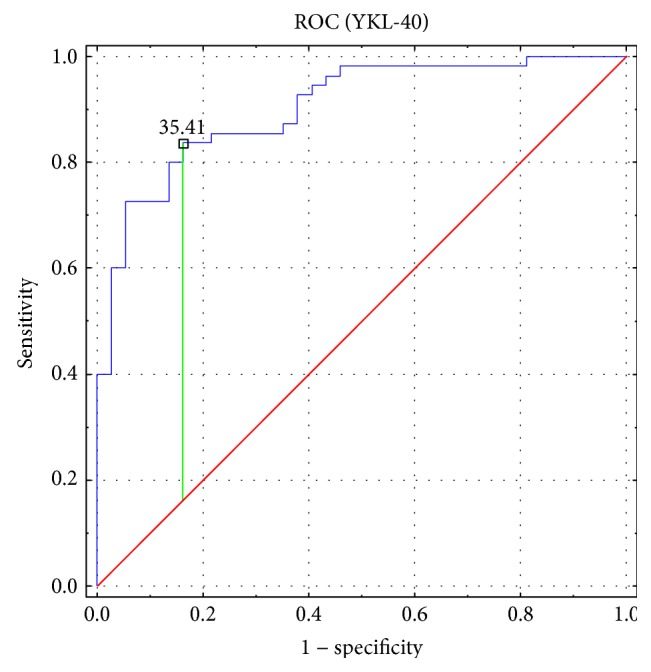
ROC analysis and the area under the curve (AUC) for YKL-40 serum level in patients with psoriasis.

YKL-40 serum concentration was significantly higher (*p* < 0.00001) in patients with psoriasis, compared to that in the control group. The mean YKL-40 serum level in the group of patients was 107 ± 77.7 ng/ml. Among the control subjects, the mean value of this parameter amounted to 25.5 ± 18.5 ng/ml. The difference in serum YKL-40 levels between the psoriatic patients and control group is presented in Figure 1. ROC analysis presented the large area under the curve (AUC) (showed in Figure 2). The optimal cut-off value for serum YKL-40 level was 35.41 with high negative (NPV) and positive predicting values (PPV) of 0.775 and 0.885, respectively.

No significant correlations were found between serum YKL-40 levels and other clinical or laboratory parameters, such as severity of skin changes, age, gender, CRP, ESR, WBC, or neutrophil count (data not shown). CRP mean value in patients with psoriasis was 4.38 ± 6.5 mg/ml and was elevated only in 11 patients, which constitutes 20% of the examined group. WBC amounted to a mean of 7.18 ± 1.8 × 10^3^/ml and was slightly above the normal ranges for only 4 patients (7.3%). All the laboratory findings within the group of psoriatic patients are summarized in Table 2.

## 4. Discussion

The presented study demonstrated that the serum level of YKL-40 in psoriatic patients is considerably elevated, and this protein may be involved in the pathomechanisms of psoriasis. The studies conducted so far to examine this matter have brought conflicting results. One of the reports has shown similar findings, as its authors reported elevated serum levels of YKL-40 in patients with psoriasis vulgaris [11]. The same paper has also presented even more elevated YKL-40 serum concentration in patients with pustular psoriasis. Serum YKL-40 in the latter group of patients was not only highly elevated but it also correlated with other inflammatory parameters, including CRP, WBC, and neutrophil count. In another study, conducted on 18 patients with arthritic psoriasis, the authors concluded that serum YKL-40 may reflect the severity of cutaneous lesions in psoriasis [12]. However, this report has some limitations. Its final conclusion was established only on the basis of a decrease in serum YKL-40 in four patients treated with infliximab, who achieved a decrease in PASI after 6 weeks of treatment. However, no correlation was observed between YKL-40 and PASI index within the whole examined group. It is worth noting that a lower concentration of YKL-40 following biological therapy could be attributed to a decrease in the systemic level of inflammation, including arthritis, rather than reflecting changes in the severity of cutaneous lesions.

Although some studies have confirmed the elevated YKL-40 serum level in patients with psoriasis, these reports focused mainly on the correlation between this parameter, the presence of endothelial dysfunction, and the risk of cardiovascular diseases in patients with psoriasis [13, 14]. One study, evaluating inflammatory markers in psoriasis, did not show any elevations in YKL-40 serum levels in psoriatic patients [15]. Another report presented the increase in YKL-40 plasma concentration in patients with psoriatic arthritis but not in patients with psoriasis without joint involvement [16]. The authors of this report did not find any correlation between YKL-40 and PASI and observed no changes in YKL-40 after the treatment of patients with psoriasis without arthritis.

Psoriasis has been known as a condition that affect not only the skin but may also predispose to systemic symptoms, such as arthritis or endothelial dysfunction. One of the possible shared pathomechanisms underlying these conditions is inflammation. Several reports have shown that YKL-40 level is considerably elevated in patients with psoriasis and other coexisting symptoms. The majority of such studies presented a significant elevation of serum YKL-40 in patients with psoriatic arthritis [16–18]. There are also reports demonstrating that YKL-40 is particularly increased in psoriatic patients with higher risk of cardiovascular disease and endothelial dysfunction [13, 14]. The associations between psoriasis and other systemic disorders, such as diabetes, gout, hypertension, and lipid profile disturbances, have been already documented; thus, it is necessary to further examine the concept of psoriasis as a systemic inflammatory disease and the role of YKL-40 protein in these complex problems [1, 19–21].

The results of this study may indicate the participation of YKL-40 in the pathogenesis of psoriasis and clearly show the elevation of this parameter in patients suffering from psoriasis without any comorbidities. However, it has to be noted that the higher level of YKL-40 may only reflect the level of systemic inflammation in psoriatic patients, as the concentration of YKL-40 does not seem to reflect directly the severity of skin changes. Moreover, it seems that in psoriatic patients, YKL-40 is a more sensitive parameter of inflammation than CRP or WBC. Within the group of our patients, CRP and WBC were elevated in 20% and 7.3% of patients, respectively. Having determined the optimal cut-off value of serum YKL-40 from the ROC curve, this parameter was elevated in 45 psoriatic patients, which constitutes 81.8% of the examined group. Presumably, the elevated level of YKL-40 may indicate psoriatic patients with higher risk of other inflammatory syndromes. It has to be taken into consideration that psoriatic patients with a higher YKL-40 level may constitute a group of patients requiring more potent treatment in order to lower the risk of systemic inflammatory complications.

The study has some limitations. It has been conducted on relatively small group of patients. It was also impossible to prove any correlation between YKL-40 levels and other parameters of systemic inflammation. The absence of correlation with the severity of psoriasis limits the clinical use of YKL-40 in monitoring the course of the disease and its treatment.

## 5. Conclusion

YKL-40 could be considered as a biomarker of inflammation in psoriasis and is more sensitive than CRP or WBC; however, the issue needs further and more detailed investigation. Increased YKL-40 may indicate psoriatic patients with a higher level of systemic inflammation, which may determine therapeutic decisions and prognosis.

## Figures and Tables

**Table 1:** Clinical characteristics of the examined group of patients with psoriasis (*n* = 55).

Parameter	Mean ± SD (range)	Minimal value	Maximal value
Age (years)	48.56 ± 13.2	18	88
Duration of psoriasis (years)	15.56 ± 15.4	0.5	60
PASI score	10.8 ± 6.8	3.8	32.1
BSA %	19.13 ± 17	2.7	80

**Table 2:** Laboratory findings in patients with psoriasis (*n* = 55).

Parameter	Mean ± SD (range)	Minimal value	Maximal value
YKL-40 (ng/ml)	107 ± 77.7	13.11	292.72
CRP (mg/ml)	4.38 ± 6.5	0.2	40.1
ESD	12.2 ± 9.6	2	57
WBC × 10^3^/ml	7.18 ± 1.8	3.82	12.14
Neutrophil count × 10^3^/ml	3.99 ± 1.3	1.78	7.75
